# Equity, diversity, and inclusion: variations in clinical and academic radiology

**DOI:** 10.1186/s13244-026-02278-5

**Published:** 2026-04-20

**Authors:** C. Kassanje, E. H. Y. Wong, J. O. Bamidele, A. C. Offiah

**Affiliations:** 1 University of Sheffield Medical School, Sheffield, UK; 2 Division of Clinical Medicine, University of Sheffield, Sheffield, UK; 3 Sheffield Children’s NHS Foundation Trust, Sheffield, UK

**Keywords:** Diversity, Equity, Inclusion, Radiological research

## Abstract

**Abstract:**

Equity, diversity, and inclusion (EDI) are fundamental to achieving fairness and representation in radiological research and practice. This review aims to examine how structural inequities related to race, sex, gender, age, disability, and socioeconomic status shape imaging research, workforce composition, and clinical outcomes.

Racial disparities persist through outdated diagnostic assumptions and unequal access to imaging, while the limited representation of minority clinicians in leadership continues to affect research priorities and inclusivity. Similarly, sex and gender inequities also remain, with women being underrepresented in academic and interventional radiology, and transgender and gender diverse individuals often excluded from research and clinical systems. These gaps highlight the importance of inclusive mentorship, equitable leadership opportunities and consistent use of inclusive terminology.

Differences in age, disability, and socioeconomic status affect participation and outcomes in imaging research. Older adults, children and people with disabilities are often excluded from imaging datasets, reducing generalisability and limiting the safe application of new technologies such as artificial intelligence. Socioeconomic inequities affect access to timely imaging and distort normative datasets, leading to misinterpretation of results in deprived populations. Inclusive recruitment, adaptive imaging protocols, and explicit consideration of social context in research design are essential to address these disparities.

To address this, radiology must prioritise inclusive recruitment, adapt imaging protocols for underrepresented groups, and integrate EDI principles into study design, dataset curation, and peer review. Embedding these practices will enhance scientific validity, ethical integrity, and patient-centred care, ensuring that imaging research truly reflects the diverse populations it serves.

**Critical relevance statement:**

This review highlights how addressing equity, diversity, and inclusion in radiological research and practice is essential for improving the relevance, accuracy, and fairness of imaging data and emerging technologies across diverse patient populations.

**Key Points:**

- Persistent gaps in diversity affect fairness within radiological research and clinical practice.
- Inequities hinder equitable representation and limit the generalisability of radiological findings.
- Inclusive practices better serve the diverse populations we care for.

**Graphical Abstract:**

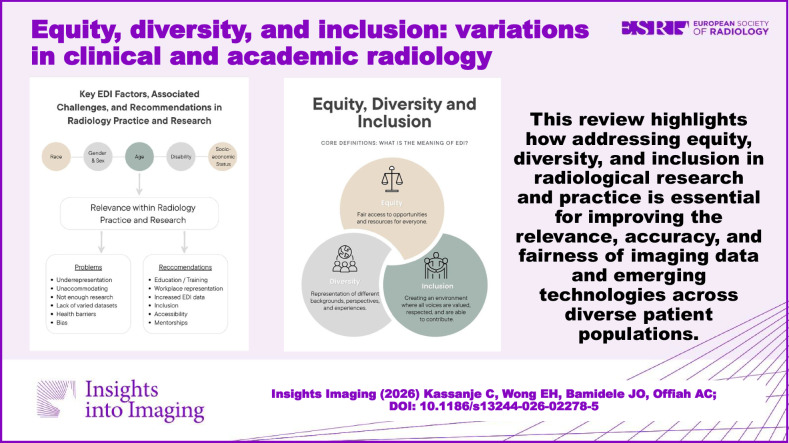

## Introduction

Equity, diversity, and inclusion (EDI) are foundational principles for ensuring fairness, representation, and belonging across all areas of society, including healthcare and medical research [1]. Diversity refers to the broad range of human characteristics, including but not limited to race, ethnicity, sex, gender, age, disability, sexual orientation, and socioeconomic status. Equity ensures that individuals with these differences are not met with unfair treatment, and inclusion fosters environments where all individuals feel respected and represented [2]. In radiology, EDI is essential for addressing healthcare disparities, improving diagnostic accuracy, and producing research that is generalisable and ethically sound [2].

In the UK, the National Institute for Health and Care Research (NIHR) Inclusion Strategy (2022–2027) highlights the necessity to better represent underserved groups in health research, particularly those who carry a high burden of disease but remain excluded from study designs, data collection, and recruitment frameworks [3]. Such exclusion not only undermines research relevance but also hinders the development of imaging criteria and findings that are accurate, inclusive, and applicable across diverse populations.

Inequities in radiology are shaped by several factors, with race, gender, age, disability and socioeconomic status standing out as key influences. These factors affect patterns of access to imaging, recruitment, leadership, training and research, ultimately shaping both patient outcomes and the inclusivity and wellbeing of the workforce. By recognising how these factors influence and have been portrayed in radiological research, it becomes possible to understand the breadth of barriers that exist and to identify where targeted interventions are most needed.

This review will explore these themes, outlining current gaps, educational considerations, and opportunities for revising imaging criteria to promote more inclusive and equitable clinical and research practice.

## Race

Racial disparities in health care are a widely recognised problem. For generations, race has been used as a key characteristic in clinical decision-making [4]. Race is a fluid social construct that divides populations based on their physical appearances and social and cultural factors. However, historically, the importance of race in medicine has been shaped by racist theories and assumptions that genetic differences between racial groups explain the variations in disease prevalence, presentation and prognosis. Such beliefs also underpinned the unethical historical experimentation on Black Americans [5]. This perspective has strongly influenced medical practice, as the concept of ‘race-based medicine’ has historically led clinicians to adjust diagnostics and treatments based on racial categories, such as in estimating glomerular filtration rate [6].

Even in radiology, a field often considered objective, historical practices demonstrate the impact of racial bias. In the 19th century, pseudoscientific ideas from the slavery era claimed that black people had increased bone density and thicker skin than white people [7]. These ideologies led X-ray technicians and radiologists to routinely administer higher radiation doses to Black and brown patients, based on unfounded assumptions about biological differences [5–9]. These practices persisted until 1968, when they were exposed as a fallacy and abandoned. This underscores how deeply race-based corrections may be embedded in clinical routines, often remaining unchallenged and unexamined.

Race-based assumptions are still present today and can directly or indirectly affect access to radiology. For instance, spirometry reference values for Black and Asian patients adjust for smaller lung capacities. This stemmed from a practice rooted in slavery-era and eugenic ideologies designed to portray that Black and Asian people have inferior lung capacities compared to their White counterparts [10, 11]. Consequently, a study found that removing race-based corrections in spirometry reclassified many Black patients into more severe categories of chronic obstructive pulmonary disease [12]. The consequences of this extend into radiology, where spirometry results often guide access to chest radiographs and CT scans for various lung diseases. Therefore, patients whose lung function has been underestimated due to race correction may be deprioritised for imaging, leading to missed diagnoses or delayed care for Black and Asian patients [9, 11]. Ultimately, demonstrating how race adjustments can propagate inequities across interconnected areas of clinical practice, including radiology.

Access to imaging remains inconsistent, with racial and ethnic minority groups across the UK and Europe facing barriers to diagnostic care. Disparities are especially pronounced in breast cancer screening. Studies show that women from Black, Asian, and minority ethnic (BAME) backgrounds in the UK have lower participation in mammography compared with White women, even after socioeconomic status and comorbidities are considered [13, 14]. These disparities are caused by cultural and religious beliefs, stigma, and low personal risk perception, alongside practical barriers such as language, limited awareness of screening programmes, and a mistrust of healthcare services [15]. As a result, breast cancer is often detected at a later stage in women from minority groups, contributing to poorer outcomes [14]. Recent reviews indicate that current interventions inadequately address the barriers experienced by ethnic communities in the UK. As mammography is often the first point of contact with radiology for many women, these barriers contribute to delayed cancer detection and create disparities in diagnostic imaging and treatment pathways [13, 14]. These disparities demonstrate that improving mammography uptake is not only a matter of public health outreach but also requires radiology services to adapt pathways and practices to ensure equitable access to imaging [15, 16]. Addressing race-based assumptions in radiology is critical for patient care and may require targeted training for clinicians on the historical and contemporary impact of racial bias, integration of race-conscious diagnostic guidelines, and routine review of imaging protocols to ensure equitable interpretation and monitoring of outcomes to ensure accountability and continuous improvement.

Racial inequities in radiology are also reflected within the workforce. The Royal College of Radiologists estimates a shortfall in consultant posts of approximately 40% [17]. These shortages exacerbate competition for senior opportunities and could reinforce barriers faced by minority ethnic radiologists. Studies highlight that racial and ethnic minorities remain underrepresented in leadership, professorships and committee positions [18, 19]. This imbalance demonstrates professional inequity and has direct implications for clinical practice, as underrepresentation of minority voices limits the diversity of perspectives that can help shape training curricula, research priorities, and policies that influence equitable patient care [17]. Furthermore, editorial and leadership structures often lack clear equity benchmarks or representation goals. An analysis of radiography journals found only a minority included EDI principles in their editorial policies, and most lacked clear guidance for inclusive language or leadership diversity [20]. This lack of structural commitment risks perpetuating cycles where underrepresented clinicians have reduced visibility and limited influence on research priorities and professional standards, reinforcing inequities within the field.

Workplace culture presents additional challenges. A European survey reported that one in six radiologists had experienced workplace discrimination, with biases often linked to gender, cultural or ethnic backgrounds [21]. These findings mirror wider NHS workforce data, where minority ethnic staff are more likely to face disciplinary action and slower career advancement, undermining both retention and progress toward equity [22]. Addressing these disparities requires more than workforce recruitment; it necessitates structural reform. Innovative interventions, such as reverse mentoring, have proven effective in bridging these gaps. A feasibility study within the Royal College of Radiologists demonstrated that reverse mentoring fosters spaces for open dialogue, mutual learning and cultural insight, enabling minority ethnic clinicians to influence leadership perspectives and organisational practices [23]. Alongside transparent progression criteria and pathways, dedicated mentorship, and formal recognition of EDI work, such schemes are essential for creating equitable opportunities within radiology [24]. To maximise impact, organisations should also implement equity-focused metrics, embed ongoing cultural competency and anti-bias training, and ensure transparent reporting of promotion and disciplinary outcomes to monitor and address systemic disparities.

## Sex and gender

Sex describes *‘a person’s assigned sex at birth’* and gender describes *‘a person’s gender identity and how they feel and perceive themselves’* [25]. Both of these categories continue to be significant barriers to equality in medicine, including radiology. Medicine has historically been a male-dominated career, but has seen improvement in recent years, with women making up approximately half or more of medical students in the USA and Europe [26]. Despite this, many specialties, particularly radiology, still show gender disparities in both staff numbers and leadership roles [26]. The recent Supreme Court judgement concluded that references to the terms ‘woman’, ‘man,’ and ‘sex’ in the Equality Act 2010 specify biological sex, defined as *‘the sex of a person at birth’* [27]. This has been met with controversy as many organisations, including medical, continue to use the binary classifications for sex interchangeably with gender, when in reality it is much more fluid and diverse [28]. Ignorance as to the distinction between these terms can lead to discrimination and poorer quality of care for patients who identify as transgender or gender diverse [25]. Acknowledging discrepancies between sex and gender is essential for organisations to implement adjustments that promote inclusive healthcare. Structured workplace training on gender diversity and communication should support this by providing practical guidance on respectful language, awareness of diverse gender identities, and strategies for fostering inclusive professional environments. Such training may help translate these principles into consistent, respectful patient care and everyday professional practice, and ensure research uses these terms accurately, as patient outcomes can differ by sex even after gender reassignment [29].

Progression throughout the medical community has led women to comprise roughly half or more of doctors in many specialties, but notable disparities remain in radiology, according to data from 29 organisations across 26 countries [30]. This information disclosed that overall, only 33.5% of these radiologists were female, and the proportions in European countries varied significantly from 29.9% in the Netherlands to 50.4% in Spain [30]. Similar statistics were displayed in other studies, with 30% of members of the European Society of Radiology being female in 2019 and 37% of female radiologists in a German radiological organisation in 2022 [26, 31]. More recent data from the Clinical Radiology Workforce Census 2024 suggest a gradual improvement, showing 42% of diagnostic radiology consultants were women, up from 36% the previous year [32, 33]. Despite this, female representation in interventional radiology remained low, at 12% in the Royal College of Radiologists and 17% in the German organisation [31, 32]. Whilst this data suggests that female representation in clinical radiology is possibly improving in recent years, it is clear that there is still a disparity in the number of female radiologists.

Several factors have been suggested to explain the gender disparity in radiology, including a male-dominated environment, discrimination, lack of work-life balance, limited patient contact, radiation concerns, reluctance towards physics and technology, and a lack of exposure during medical school [30, 34]. However, literature suggests that the most significant demotivators are the lack of female role models and mentors in the speciality [35]. Mentoring may be particularly beneficial for women, as unconscious biases from colleagues or panels and personal reservations can make applying for roles more challenging [26]. The absence of women in senior roles limits the integration of female perspectives into service design and patient management and reduces opportunities for mentorship that can inspire other women to pursue leadership positions, thereby reinforcing systemic underrepresentation. Furthermore, a study found that mentoring by women increased the proportion of female first authors in European radiology journals, although data from these journals remain scarce [36]. The study also found that female last authors, who represent female senior researchers acting as coordinators, increased at a slower rate than the number of first authors, suggesting that fewer women are holding senior positions [36]. Additionally, in 2020, only 20% of European Society of Radiology chairs were women, with the theorised reasoning aligned with the previously mentioned barriers [35].

The presence of women within organisations has proven to be beneficial to an array of workforces, through the diversity provided in skills, viewpoints and critical thinking [26]. It has also been noted that there is a positive association between female presence in radiology and the Gender Development Index (GDI) [30]. This is a metric devised by the United Nations to quantify equality between women and men in health, knowledge, and living standards, where a higher number indicates less disparity [30]. When observed in various radiological organisations in several countries, it was concluded that those with higher proportions of female radiologists had higher associated Gender Development Index values, suggesting that female representation in radiology has positive effects on equality between both women and men in many aspects of life [30]. It is therefore reasonable to suggest that it is in the best interest of medical specialties, including radiology, to take measures to increase female representation. This could include formal mentoring programmes, increased leadership courses, mandatory unconscious bias training, gender quotas and targeted outreach to encourage female participation [26]. Addressing retention is also equally important, as women often face a disproportionate burden of family responsibilities that can hinder work–life balance. Initiatives such as accessible childcare support and flexible working policies may help mitigate this barrier [18]. While not foolproof, implementing one or more of these strategies could improve access for women into radiology and promote a more balanced workforce.

Whilst improvements must be made to increase access for women in radiology, it is imperative that we also acknowledge the inequality faced by transgender and gender-diverse (TGD) individuals [28]. TGD is a general term referring to anybody who has a gender identity different from their assigned sex at birth and covers a wide breadth of diverse, fluid gender identities [28, 37]. However, research in radiology often fails to take this into account, frequently using terms such as ‘gender diversity’ to discuss the discrepancies between women and men or even using “gender” when referring to newborn children [28]. In one of the studies investigating gender disparity within interventional radiology, participants were required to fill out a questionnaire in which they were asked about their gender [31]. The study reported that three individuals specified their gender as ‘other’ and, as a result, were excluded from the study to ‘ensure anonymity’ [31]. Another study determined authors from radiology articles to be female or male based on their first names, excluding any authors whose binary gender could not be determined [36]. The exclusion of diverse gender identities in research and education reinforces ignorance and bias, resulting in poorer interactions and outcomes for TGD individuals [28]. Studies suggest that TGD students and doctors make efforts not to disclose their gender identities due to the non-inclusive environments [28]. This can be professionally and personally harmful, particularly when organisations fail to implement changes that support participation and inclusivity. Recruitment and retention of TGD health care providers should be prioritised to increase awareness and promote a more informed, inclusive work environment [28]. In addition, inclusive guidelines, accurate recording of sex and gender, and targeted educational reforms are needed to address gaps in knowledge and reduce the associated health disparities seen in staff and patients who often experience discrimination and microaggressions [38].

Although race and gender represent distinct demographic dimensions, their intersection can compound barriers to representation, particularly for women from minority ethnic backgrounds. Addressing these inequalities, therefore, requires an intersectional perspective that invites further consideration of how age, disability, and socioeconomic status shape experiences within radiology.

## Age, disability, and socioeconomic status in imaging

A truly inclusive approach to EDI must reflect the full spectrum of identity and lived experience, particularly among groups historically underrepresented in research [3]. Globally, EDI initiatives predominantly focus on gender and race equality within healthcare policy and interventions [39]. In the UK, this trend continues, while other characteristics, such as disability, age, sexual orientation, socioeconomic status, and religion, receive comparatively less attention in research [39, 40]. Broader EDI efforts in radiology must turn to these overlooked dimensions of identity and aim to close potential gaps in care and research, recognising that, alongside race and gender, they interact to create layered inequities in access, participation, and career progression.

## Age

Despite age influencing anatomy, physiology, and imaging interpretation, paediatric and older adult populations remain disproportionately underrepresented in research. Older adults are repeatedly excluded from clinical trials, and paediatric patients are infrequently included in large-scale imaging datasets [41–43]. A review of 53 randomised controlled trials (RCTs) on non-invasive chest pain imaging found a participation-to-prevalence ratio of 0.21 for those ≥ 75 years versus 2.13 for those under 65, highlighting severe underrepresentation of older adults [44]. In the UK, over 40 leading funders and charities, such as Age UK and the British Geriatrics Society, are committed to increasing the inclusion of older adults and those with multimorbidity into clinical research, deeming their current exclusion both unethical and unrepresentative [45]. Supporting this, a study found that most professionals believe that age-based exclusion is unjustified and that such practices create problems for clinicians and disadvantages for patients [46]. Older adults endure the greatest burden of disease and utilise imaging modalities more than any other demographic [41, 47, 48]. Excluding older adults from research limits generalisability and allows important age-specific considerations to go unrecognised. For instance, frailty and degeneration can produce vague or non-specific imaging findings, making it difficult to distinguish between normal age-related changes and disease [49, 50]. However, through research inclusion and customised imaging protocols based on frailty, a holistic approach can be taken to avoid unnecessary procedures, manage minor incidental findings appropriately, and ensure atypical or age-related presentations are recognised to prevent interventions that may cause more harm than benefit [49, 51].

In paediatric radiology, standardised diagnostic reference levels (DRLs) and age-appropriate imaging criteria are well established [52, 53]. Initiatives such as adherence to the ‘as low as reasonably achievable’ (ALARA) principles and DRLs have supported safer imaging practices, and research activity in paediatric radiology continues to steadily increase across Europe [54, 55]. Despite this growing engagement, there remains a notable lack of representation in large‑scale imaging datasets, with the UK Biobank choosing to explicitly exclude paediatric patients [43]. As artificial intelligence (AI) tools and large-scale imaging repositories develop, the underrepresentation of paediatric data limits innovation and may lead to biased outcomes when applied in paediatric settings [56]. The lack of paediatric-specific datasets also hinders the ability to train, validate, and deploy AI systems tailored to the unique physiological and developmental characteristics in children. Without diversified dedicated data, AI models are likely to be inaccurate, incomplete and underperform in paediatric applications, particularly in tasks such as disease classification and image segmentation [56, 57]. To advance clinically robust and safe AI for children, future development must prioritise age-specific training data, validation across diverse paediatric subgroups, and rigorous evaluation of performance metrics tailored to younger populations. Until appropriate frameworks and age-diverse datasets are established, the clinical use of AI in paediatric imaging should proceed with caution, by recognising the current limitations and the potential for errors.

## Disability

Disability, as defined by the UK Equality Act 2010, refers to a physical or mental impairment that substantially affects a person’s long‑term ability to carry out normal day‑to‑day activities [58]. An estimated 16% of the world’s population, one in six people, live with significant disabilities and often face serious health inequities [59]. For instance, individuals with disabilities sometimes face challenges such as difficulty maintaining positioning or remaining still during imaging procedures, leading to motion artefact with suboptimal image quality, and in some cases, images unsuitable for clinical interpretation [60, 61]. Physical accessibility in radiology also extends beyond formally recognised disabilities, as comparable technical and logistical barriers can affect patients with larger body habitus, through equipment limitations and positioning difficulties that complicate image acquisition [62]. These issues increase the likelihood of repeat imaging, unnecessary radiation exposure, and potentially inaccurate diagnoses [60, 61]. In the absence of adapted protocols and inclusion in research and diagnostic standards, such disparities risk becoming embedded in routine practice [63, 64]. One study suggests that disability competence and awareness training, along with interdisciplinary collaboration and sensory-friendly modifications, are essential, especially for those with sensory or cognitive impairments [65]. In addition, to address barriers faced by patients with larger body habitus, improvements in equipment design and technological innovation, including AI, are needed to accommodate all patients and enhance diagnostic quality [62].

Neuroimaging research has increasingly been used to investigate neurodevelopmental conditions such as autism spectrum disorder, making significant progress in identifying structural and functional brain differences [66–68]. However, this topic continues to grapple with heterogeneity in findings and the limited inclusion of diverse populations [68]. A meta-analysis found that many studies systematically excluded or under-represented autistic individuals with severe intellectual disability, comorbidities, or lower IQ, thereby limiting the generalisability of findings to the broader spectrum and marginalising those with higher support needs. Several studies also relied on small sample sizes, further reducing statistical power and representativeness [68]. One study underscored that reliance on binary diagnostic categories in imaging datasets, such as labelling individuals as having or not having autism spectrum disorder, oversimplifies the complexity of the condition and restricts the development of models capable of capturing the full range of autistic characteristics [69]. Additionally, while the use of anonymised imaging datasets can be ethically necessary, they often lack the contextual detail needed to support generalisable, inclusive models, further limiting their relevance to clinical practice [70, 71]. To address these concerns, the use of larger, more inclusive samples is essential to better reflect the diversity of the autistic population and support more accurate characterisation of the disorder [68].

With the rapid growth of innovation and AI in radiological research, we can no longer afford to continue with narrow design perspectives that exclude the full spectrum of disability, as such approaches risk perpetuating biased and discriminatory outcomes for underrepresented individuals [71]. Ensuring that emerging technologies are developed with accessibility and inclusivity in mind will be critical to preventing the reinforcement of existing inequities in radiological care.

## Socioeconomic status

Socioeconomic status describes an individual’s or group’s social and economic standing, through indicators such as income, educational attainment, and occupation [72]. While socioeconomic status has long been associated with health disparities, its impact on diagnostic imaging, both in clinical practice and research, has gained increasing attention. These disparities not only influence who receives imaging but also how findings are interpreted, the generalisability of data, and the validity of research conclusions. Recent UK-based research has demonstrated that differences in socioeconomic status are associated with structural differences in the brain that manifest as identifiable and measurable imaging abnormalities [73]. Additionally, a UK Biobank study of nearly 40,000 individuals examined how socioeconomic status moderated the connection between depression severity and cortical brain volumes [74]. Initially, cortical regions appeared to be negatively associated with depression; however, after statistically adjusting for socioeconomic status, most of these associations lost statistical significance [74]. A further study of approximately 10,000 individuals found widespread associations between socioeconomic status and both grey and white matter across numerous brain regions. Higher socioeconomic status was linked to increased volume and structural integrity in the left hemisphere, while lower socioeconomic status was more associated with the right [75]. This systematic lateralisation was observed across diverse socioeconomic status indicators, suggesting that socioeconomic conditions may shape brain development and cognitive architecture in patterned and functionally relevant ways [75]. Overall, these findings highlight the critical role of socioeconomic status in brain health and mental illness, underscoring the need to record it in imaging research to avoid misleading conclusions.

While the structural effects of socioeconomic status are increasingly well documented, an additional concern is the use of normative imaging datasets in clinical and research contexts. UK Biobank, despite its scale and utility, is acknowledged to be demographically skewed toward healthier, more affluent, and better-educated participants [76]. Subsequently, normative thresholds derived from this cohort may not be appropriate for more deprived populations, due to the chance of radiological findings being misinterpreted as pathological rather than reflecting normal variation underrepresented in existing datasets. To address this, imaging criteria and normative standards should be actively revised to incorporate socioeconomic diversity, ensuring more accurate and equitable interpretation in both clinical and research settings.

Beyond these challenges in image interpretation, access to timely and appropriate imaging remains an ongoing issue. These radiology-specific disparities reflect broader societal patterns, as individuals with lower socioeconomic status are more likely to experience poorer health due to factors such as substandard housing, inadequate nutrition, and limited access to preventive care [72]. Despite the NHS’s universal diagnostic services, patients from lower socioeconomic backgrounds often experience delayed or less appropriate imaging, particularly for neurodegenerative conditions such as dementia and Parkinson’s disease, even where referral guidelines exist [77]. This is partly attributed to complex factors, including variability in primary care referral patterns, differences in health-seeking behaviours, and possible implicit biases among healthcare providers [77]. Consequently, patients from deprived areas often present with more advanced disease, underscoring the need to address socioeconomic disparities in imaging access and interpretation. Training should address social determinants of health and biases affecting imaging, equipping clinicians to ensure equitable diagnostic pathways and consider socioeconomic factors in clinical decision-making.

## Recommendations

Evidence from healthcare and radiology demonstrates that compound disadvantage, for instance, being from a lower socioeconomic background, female, and from a minority racial group, is associated with poorer health outcomes, reduced access to care, and systemic underrepresentation in research [72]. To address these inequities, radiology must adopt coordinated, intersectional strategies across research, clinical practice, workforce development, and technological innovation.

1. Adopt an Intersectional Approach: 
  This can be achieved through multidimensional frameworks in clinical practice, research, and workforce planning, with ongoing policy review to prevent unintended disadvantages for specific groups.
2. Improve Workforce Representation and Leadership Diversity: 
  Strategies include mentorship, targeted recruitment, and transparent promotion pathways. Visible role models among women, minority ethnic, and transgender or gender-diverse staff could further inspire participation, leadership, and inclusivity in radiology, ultimately strengthening trust and inclusivity in the profession.
3. Ensure Inclusive Participation in Imaging Research and Datasets: 
  Studies should intentionally include participants across all ages, functional abilities, socioeconomic backgrounds, and other underrepresented groups, while recording detailed demographic information. Extra consideration should be given to populations often excluded, such as older adults, paediatric patients, individuals with disabilities, and socioeconomically deprived communities. Inclusion ensures that imaging norms, AI algorithms, and diagnostic criteria accurately reflect the diversity of the patient population.
4. Adapt Training and Imaging Protocols for Accessibility: 
  Integrating patient factors into study and service design, with staff training in disability, cultural, gender, and transgender awareness, is important. Alongside this, adaptable equipment and imaging protocols can ensure participation, comfort, and diagnostic quality for all patients, including those with physical disabilities, larger body habitus or other specific individual needs.
5. Address Socioeconomic Barriers: 
  Radiology services should use targeted outreach, tailored pathways, and clinician education on social determinants of health to support equitable imaging access and reduce diagnostic disparities.

## Conclusion

For research to meaningfully inform clinical practice, it must reflect the diversity of the population it aims to serve. Without the active inclusion of underrepresented groups, radiology risks producing evidence that is biased towards the majority and unrepresentative of marginalised populations. Achieving genuine equity in imaging, therefore, requires not only recognising these disparities but also ensuring that minorities are actively recruited, represented, and prioritised in research design and participation. It also necessitates a critical reassessment of imaging criteria and current standards to reflect a broad range of demographic and social contexts, thus improving accuracy and reducing misinterpretations across populations. Peer reviewers should likewise assess manuscripts through an EDI lens.

Taken together, narrowly focused analyses risk overlooking structural determinants of health that shape clinical outcomes. Meaningful progress will require the deliberate inclusion of underrepresented groups, supported by continuous innovation, inclusive education, and a sustained commitment to equity in radiological research and practice.

## Data Availability

No original datasets were generated or used within this educational review. Therefore, data sharing is not applicable.

## Abbreviations

AI: Artificial intelligence
DRLs: Diagnostic reference levels
EDI: Equity, diversity, and inclusion
TGD: Transgender and gender-diverse
